# The relationship between sexual violence and human immunodeficiency virus (HIV) infection among women using voluntary counseling and testing services in South Wollo Zone, Ethiopia

**DOI:** 10.1186/1756-0500-6-271

**Published:** 2013-07-15

**Authors:** Fatuma Hassen, Ngussie Deyassa

**Affiliations:** 1 School of Medical Laboratory Science, Addis Ababa University, Addis Ababa, Ethiopia; 2 School of Public Health, Addis Ababa University, Addis Ababa, Ethiopia

## Abstract

**Background:**

Gender based violence affects the health and wellbeing of women across the world on an epidemic scale. While women remain more vulnerable to both sexual violence and risk of HIV infection, they are less able to access health and other welfare services than men. These vulnerabilities are further compounded by social factors, including the low status of women in many communities and their lack of decision-making power, both within the household and in wider society. The objective of this study was to assess the relationship between sexual violence and HIV infection among clients of voluntary counseling and testing (VCT) services in South Wollo Zone, Ethiopia.

**Methodology:**

A facility based cross sectional study was conducted using quantitative methods on a sample of 647 people living in seven selected districts of South Wollo Zone, Amhara Regional State.

**Results:**

The study revealed that sexual violence is significantly associated with the risk of HIV infection. The prevalence of lifetime sexual violence, lifetime partner violence, and last 12 months partner violence were 34.6%, 32.3% and 10.5% respectively. Both partner violence and lifetime sexual violence by another perpetrator were associated with HIV. The overall prevalence of HIV among VCT users was 21.5%. Both before (crude analysis) and after the results were adjusted for selected variables, women who experienced sexual violence in the last 12 months by their intimate partner or by another perpetrator is significantly associated with their HIV status. The chances of having HIV was 1.97 times higher among women victims who have a history of lifetime partner violence when compared with women who are not victims; crude odds ratio (COR) = 1.97, 95% Confidence Interval (CI), (1.34 - 2.90).

**Conclusion:**

The study revealed that sexual violence is significantly associated with the risk of HIV infection. Empowerment of women can be used as an important tool to reduce both sexual violence and HIV. More importantly policy issues must be set by all actors to take action on the mediating variables that interacted with violence to aggravate the transmission of HIV.

## Background

Violence against women includes any act of verbal or physical force, coercion or life-threatening deprivation, directed at an individual woman or girl that causes physical or psychological harm, humiliation or arbitrary deprivation of liberty and that perpetuates female subordination The most pervasive form of gender violence is abuse of women by intimate male partners. A recent review of 50 population-based studies carried out in 36 countries indicates that between 10 and 60% of women who have ever been married or partnered have experienced at least one incident of physical violence from a current or former intimate partner [1].

Gender based violence and human immunodeficiency virus (HIV) infection are problems of great public health worldwide, especially sub-Saharan Africa and much of the developing countries. This is due to their far reaching social, economic and public health consequences. The two problems have gender inequality and gender power imbalances as the driving force behind the “epidemics”. HIV infection is mainly acquired through heterosexual relations, which themselves are greatly influenced by socio-cultural factors, underlying which are gender power imbalances [2].

Gender-based violence is identified as a significant driver of HIV infection in women. Violence increases the risk of HIV infection in women due to physiological and psychological reasons. Uninfected women are about twice likely to contract HIV from infected men than the other way round. Biologically, women are more vulnerable to infection and forced sex than men. This further increases the risk of HIV transmission to women due to tears and lacerations, especially in adolescent girls. Even threat of violence can have serious negative consequences. Women, who fear violence, are less able to protect themselves from HIV infection. They do not have the power to negotiate the circumstances in which sex takes place and the use of condom, to refuse unwanted sex, to be tested for HIV and to seek treatment after infection. Women report that they fear discrimination, physical violence, and rejection by their respective families if they disclose their HIV-positive status [3].

Physical and sexual intimate partner violence (IPV) and childhood sexual abuse (CSA) increase the risk of HIV infection both directly via viral transmission and indirectly, through increasing the likelihood of subsequent risky sexual behavior [4].

Based on WHO “Multi-country study on Women’s Health and Domestic Violence against Women” study there is a direct link between gender-based violence and HIV infection, particularly in young women. This study, found that, in countries such as Bangladesh, Ethiopia, Tanzania and Peru, violence in the home is widespread and ranges upwards to just over 70% [5]. Gender-based violence and gender inequality are increasingly cited as important determinants of women's HIV risk [6].

Recent studies across the African continent have yielded prevalence estimates of either physical or sexual IPV ranging from 18% in the past year to 71% in a lifetime. IPV has also been linked to HIV, especially in regions with a high prevalence of seropositivity. In a sample of 245 women presenting for voluntary counseling and testing (VCT) in Dar es Salaam, Tanzania, those found to be infected with HIV were 65% more likely to have been in a physically abusive relationship in their lifetime, and more than twice as likely to be in a currently abusive relationship [7].

A study conducted among women attending antenatal care in Soweto, South Africa, indicated that women who had experienced partners’ violence and controlling behavior are nearly 1.5 times more likely to contract HIV than those who do not [8].

Women, especially young women, are not in a position to convince their partners use condom. In this and in other forms of protection measures their control and decision making power is minimal. Their access to health and social services is also less. In addition, as a study revealed in Uganda many women were found unwilling to access available HIV/AIDS services for fear of being physically assaulted or threatened by their husbands or partners [9].

In 2010, approximately 1.2 million Ethiopians were living with AIDS. Of which HIV prevalence is higher among women (1.9%) than in men (1.0%). According to 2011 Ethiopian HIV/AIDS health profile, adult HIV prevalence was estimated at 1.5%. In urban areas, women are more likely to be infected than men (5.2% and 2.9%) respectively. Both gender based violence and substance abuse are factors that exacerbate the spread of HIV among certain groups of women [10].

In South Wollo zone HIV prevalence is high and this cross sectional study was conducted to assess the relationship between sexual violence and HIV infection among women attending VCT services in the area.

## Results

Out of 667 study participants, 647 of them participated at different sites, making the response rate 97%. On average, about 80 respondents (range between 60 and 100) were included from each VCT center. The total number of participants varied for each VCT center because it was determined by the flow of clients in each health facility. The mean age of the study subjects, who attended VCT centers, was 25.7 years and 284 (43.9%) were young women between the ages of 15-24 years. More than half (52.7%) of the women were married or cohabiting with a partner; a significant number 228 (35.2%) were illiterate. Religion wise 407 (62.9%) of the women were Muslims and ethnically 611 (94.4%) were Amhara. A total of 275 (42.5%) of the respondents were housewives, though there were high proportions of students and farmers. Approximately half (47.0%) of the respondents were from an urban population. The median age at first sexual intercourse was 16 years with the majority of the respondents (70%) reported that they had had sexual intercourse before they reached 18 years of age.

About half, 327 (50.2%) of the respondents had only one partner, and about a similar proportion 320 (49.5%) of them had multiple (two or more) partners. The study also revealed, 114 (17.6%) of the subjects had an extra-partner during the last 12 months. Similarly, 10% of the respondents had a history of sexually transmitted infection. Besides, 232 (35.9%) of the study participants reported they chew khat and 86 (13.3%) of participants drink alcohol.

Prevalence of HIV among VCT users in South Wollo zone was 21.5%, with 95% confidence interval (CI) (18.34% - 24.6%). The overall lifetime prevalence of at least one form of sexual violence by any perpetuator was about 36.3%, with 95% CI (32.6% - 40.0%).

Similarly, of the total 647 respondents, who had ever had a partner, the prevalence of lifetime sexual violence by an intimate partner was 32.3%, with 95% CI (28.7% - 35.9%). The prevalence in the last 12 months prior to the date of the survey was 10.5%, with 95% CI (8.14% - 12.8%). Lifetime prevalence of experiencing sexual violence by a stranger is 10.2% with (95% CI), (7.9 and 12.5%); this prevalence in the last 12 months was 3.4% (95% CI 2.01%-4.79%) (Table 1).

**Table 1:** Prevalenzce of HIV test status and experiencing of sexual violence in lifetime and last 12 months in South Wollo Zone women VCT users, June 2009, (n =647)

**Category**	**Prevalence**	**95% CI**
Sexual violence		
By intimate partner		
Life time	32.3	(28.7 – 35.9)
Last 12 months	10.5	(8.1 – 12.9)
By stranger		
Life time	10.2	(7.9 – 12.5)
Last 12 months	3.4	(2.0 – 4.8)
Before 15 years of old	5.6	(3.8 – 7.4)
Ever by any perpetuator	36.3	(32.6 – 40.0)
HIV status	21.5	(18.3 – 24.6)

NB.

*HIV* Human Immune Virus.

*CI* Confidence Interval.

*VCT* Voluntary counseling and Testing.

Table 2 presents the socio demographic correlates of HIV status and life time violence among women of VCT users in South wollo zone. Similarly, Table 3 shows, the comparison of HIV status and life time sexual violence by risky sexual behavior and substance use.

**Table 2:** Socio-demographic correlates of HIV status and life time violence among women VCT users in South Wollo, June 2009, (n=647)

**Character**	**Sample**	**HIV preva.**	**Crude OR (95% COR)**	**Violence prevalence**	**Crude OR (95% COR)**
**Age group**					
15-24 yrs	284	(10.2)	1.00	(32.7)	1.00
25-34 yrs	244	(30.7)	**3.90 (2.43–6.24)**	(40.6)	1.40 (0.98–2.00)
35+ yrs	119	(29.4)	**3.66 (2.11–6.35)**	(36.1)	1.16 (0.74–1.82)
**Marital status**					
Never married	110	(11.8)	1.00	(35.5)	1.00
Married /cohab.	341	(17.6)	1.59 (0.83–3.02)	(28.7)	0.73 (0.46–1.15)
Widow/divorce	196	(33.7)	**3.70 (1.97–7.25)**	(50.0)	**1.82 (1.12–2.94)**
**Educational status**					
Illiterate	228	(22.8)	1.25 (0.73–2.19)	(43.0)	**2.13 (1.37–3.31)**
Elementary	262	(21.8)	1.18 (0.70–1.99)	(36.6)	**1.63 (1.05–2.53)**
Sec. & above	157	(19.1)	1.00	(26.1)	1.00
**Religion**					
Christian	240	(22.5)	1.04 (0.69–1.55)	(33.3)	1.00
Muslim	407	(21.5)	1.00	(38.1)	1.23 (0.88–1.71)
**Ethnicity**					
Amhara	611	(21.1)	1.00	(36.0)	1.00
Non-Amphara	36	(27.8)	1.43 (0.67–3.05)	(41.7)	1.26 (0.64–2.51)
**Occupation**					
House wife	275	(22.5)	1.00	(32.4)	1.00
Gov temp.	46	(30.4)	1.50 (0.75–2.99)	(37.0)	1.22 (0.64–2.34)
Student	91	(6.6)	**0.24 (0.10–0.58)**	(23.1)	0.62 (0.36–1.08)
CSW	9	(33.3)	1.71 (0.41–7.06)	(77.8)	**7.31 (1.48–35**.**9**)
Farmer	111	(15.3)	0.62 (0.34–1.12)	(43.2)	**1**.**59** (**1**.**01**–**2**.**50**)
Other	115	(32.2)	**1**.**63** (**1**.**00**–**2**.**64**)	(46.1)	**1**.**78** (**1**.**14**–**2**.**78**)
**Resident**					
Urban	304	(28.9)	**2**.**33** (**1**.**58**–**3**.**43**)	(36.5)	0.98 (0.71–1.35)
Rural	343	(14.9)	1.00	(36.2)	1.00

**NB**. In the above table (Table 2), there is difference comparator category for religion, this is because the prevalence of HIV was lower among Muslim, we use Muslim as a comparator for calculating the odds ratio for HIV. On the other hand since the prevalence of violence was lower among Christian, therefore we use Christian as a comparator to calculate crude odds ratio for violence.

NB.

*HIV* Human Immune Virus.

*CI* Confidence Interval.

*COR* Crude Odds Rati.

*OR* Odds Ratio.

*VCT* Voluntary counseling and Testing.

*The bold face is used to show significant statistical association between Socio-demographic correlates of HIV status and life time violence.

**Table 3:** **Comparison of HIV status and life time sexual violence by risky sexual and substance use behaviors**, **of South Wollo Zone women VCT users**, **June 2009** (**n**=**647**)

**Character**	**Sample**	**HIV prevale**	**Crude OR**	**Violence prevale**	**Crude OR**
**Age at first intercourse**					
< than 18 yrs	453	(21.9)	1.08 (0.70–1.66)	(38.2)	**1**.**84** (**1**.**26**–**2**.**70**)
18 and above yrs	194	(20.6)	1.00	(32.0)	1.00
**No. of casual partner**					
One	327	(16.2)	1.00	(27.2)	1.00
Multiple	320	(26.9)	**1**.**90** (**1**.**29**–**2**.**78**)	(45.6)	**2**.**18** (**1**.**55**–**3**.**06**)
**Extra**-**partner sex**					
Yes	114	(21.2)	1.09 (0.67–1.78)	(34.5)	**1**.**53** (**1**.**01**–**2**.**31**)
No	533	(22.8)	1.00	(44.7)	1.00
**History of STI**					
Yes	65	(50.8)	**4**.**63** (**2**.**72**–**7**.**86**)	(52.3)	**2**.**07** (**1**.**24**–**3**.**48**)
No	582	(18.2)	1.00	(34.5)	1.00
**Khat chewing**					
Yes	232	(26.7)	**1**.**60** (**1**.**09**–**2**.**34**)	(44.4)	**1**.**71** (**1**.**22**–**2**.**38**)
No	415	(18.6)	1.00	(31.8)	1.00
**Alcohol drink**					
Yes	86	(29.1)	1.60 (0.96–2.67)	(45.3)	1.54 (0.97–2.44)
No	561	(20.3)	1.00	(34.9)	1.00

NB.

*HIV* Human Immune Virus.

*CI* Confidence Interval.

*OR* Odds Ratio.

*STI* Sexually Transmitted Infection.

Khat- An evergreen shrub, having dark green leaves that is chewed for their stimulating effects.

*The bold face is used to show significant statistical association between risky sexual and substance use behaviors with HIV status and life time sexual violence.

HIV prevalence is significantly higher among women who experienced violence. This is true both crudely, and after results were adjusted for marital status, occupational status, number of partners ever, khat chewing habit and residence (Table 4).

**Table 4:** **Association of different forms of sexual violence with HIV**, **crude and adjusted OR**, **after controlling for selected variables**, **June 2009**, (**n**=**647**)

**Sexual violence**	**Sample**	**HIV + %**	**Crude**	**Adjusted**
			**OR (95% CI)**	**OR (95% CI)**
By intimate partner				
Life time				
No	438	(17.6)	1.00	1.00
Yes	209	(29.7)	**1**.**97** (**1**.**34**–**2**.**90**)	1.4 (0.90–2.10)
Last 12 months				
No	579	(19.5)	1.00	1.00
Yes	68	(38.2)	**2**.**55** (**1**.**50**–**4**.**33**)	**2**.**4** (**1**.**40**–**4**.**40**)
By any perpetuator				
No	412	(17.2)	1.00	1.00
Yes	235	(28.9)	**1**.**95** (**1**.**33**–**2**.**86**)	**1**.**4** (**1**.**00** –**2**.**10**)

**NB:** The logistic regression model is adjusted for women marital status, number of partners, history of STI during last 12 month and chat chewing habit.

NB.

*HIV* Human Immune Virus.

*CI* Confidence Interval.

*OR* Odds Ratio.

*The bold face is used to show significant statistical association between different forms of sexual violence with HIV.

## Discussion

This study is conducted across seven districts in ten VCT centers of South Wollo zone. It covered a wide area of the Zone. The response rate of the study is high (97%), this may be due to the training given and the quality of supervision carried out in the area. The proportion of widow/divorced women in the study is about 30.3%, which is relatively higher than the normal population distribution of the Demographic Health Survey of Ethiopia (EDHS 2005) 10.6% [11]. This could be attributed to the fact that most widowed or divorced women are forced to check their HIV status, as they are perceived to be more at risk. It may also be due to the prevalence of early marriage, which often ends in divorce. An equal proportion of respondents from rural and urban areas were included. The majority (70%) of the respondents started sex before they reached 18 years of age. The median age at first sexual intercourse is 16 years, which is comparable with the regional average (15.5 years) [12].

The overall prevalence of HIV among women of VCT users in South Wollo zone is 21.5%. This prevalence is higher when compared with the region’s DHS and Antenatal Care Survey which was 1.8% and 4.5%, respectively. But this study’s finding is lower than the study done in Addis Ababa that estimates 24.5% [12]. In this study, the prevalence of violence is 36.3%. This is lower than the study conducted in Butajira district, central Ethiopia, which was 59% [13]. The reason for this difference could be attributed to methodology: the current study was a facility based survey whereas the Butajira study was a community based study. According to the current study, the lifetime risk of forced sex by a stranger is 10.2%. This is lower than a study conducted among VCT clients in Addis Ababa (14.9%) [14].

The prevalence of sexual abuse during childhood is 5.6%. This figure is comparable with a study done among pregnant women in Soweto, South Africa which the estimated prevalence was at 8% [8].

A variation in HIV/AIDS prevalence is also observed by marital status, which is significantly higher among divorced or widowed women. This is comparable with the 2005 Ethiopian Demographic Health Survey (EDHS) and a study of the socio-demographic profile and prevalence of HIV among VCT clients in Addis Ababa [11,13].

One key finding of this study is that it demonstrates the higher prevalence of HIV among illiterate women when compared to those who completed secondary education or above. Compared to a study done among VCT clients in Addis Ababa [14], the result obtained in this study can be attributed to, specifically, to low information dissemination on HIV and, in general, the fact that illiterate women are less empowered to protect themselves from the risk of violence [11].

The prevalence of both HIV and sexual violence did not differ significantly by other background variables such as religion or ethnicity. In connection to occupation, a significantly higher HIV prevalence and sexual violence was reported among commercial sex workers. The prevalence of HIV is higher among urban respondents when compared with rural respondents (28.9% versus 14.9%). This is in agreement with EDHS 2005 (7.7% versus 0.6%). The prevalence of HIV is significantly higher among those who reported risky behavior such as multiple partners, history of STIs, substance abuse (khat and alcohol). This is in agreement with the study conducted on the association between substance abuse and HIV infection among people visiting HIV counseling and testing centers in Addis Ababa [15]. In addition, the finding of this study is similar with the finding of the study conducted on racial and ethnic minority women in the United States. In line with this, those who had more sex partners had more STIs, and more severe history of physical and sexual trauma [16].

Another study conducted in South Africa also found that experience of violence and controlling behavior from male partners was strongly associated with increased risk of HIV infection among women. This study also noted that multiple partners, transactional sex, and substance abuse increased HIV risk among women [17].

In this study, most risk behavior variables have significant association with both HIV and sexual violence. The prevalence of violence was higher among the confounding variables such as women with multiple partners, women chewing chat and women with STI. Since khat is a substance/ drug which can stimulate behaviors, it may have confounding effect on violence. Similarly, those women having multiple sexual partners may have more chance of being violated as compared with those having a single partner each. This study is supported by a similar study which showed that adolescents who were reported as high level drug users at a time also had more sexual partners, and also practiced higher frequencies of unprotected sex [18].

Risky sexual activities include inconsistent condom use, having multiple sexual partners over one’s lifetime, or having intercourse with a casual partner. Studies conducted to date indicate that drinking alcohol and taking drug illicit and often occur in association with risky sexual activity [19]. Such an interaction, of these confounding variables, in the causal chain clearly calls the attention of policy makers to take the necessary action on these issues to eliminate sexual violence from the community and at the same time reduce the prevalence of HIV/AIDS.

The chance of HIV and violence was higher among women with multiple lifetime partners, which is similar to the EDHS results [11]. Women who are experiencing partner violence are at an increased risk of HIV infection. This is similar to an urban study done on minority African, American and Latin American women who attend health care clinics for the treatment of such infections [20].

This study has shown that, women which have life time history, of sexual violence have 1.97 times at higher risks of acquiring HIV. Similarly, there is a significant relationship between HIV infection in the last 12 months, and sexual violence by perpators. This is comparable with a study conducted in South Africa, which revealed that increasing frequency of physical and/or sexual violence was associated with increased odds of HIV infection whether or not psychological abuse was also reported [8].

To see the effect of different forms of violence on HIV transmission and prevalence, a logistic regression model was fitted with background variables and risky behavior variables. After controlling for selected variables (which have significant association in crude odds ratio) in the last 12 months partner violence and violence by any perpetrator remain significantly associated with HIV status. In this study, the risk of HIV infection is 2.45 times higher among those who have a history of partner violence during the last 12 months. This is comparable with a study conducted in Tanzania, which revealed that HIV-positive women were over 2.5 times more likely to have experienced violence by their partner than other women [21].

As shown in the result, women who experienced sexual violence, by an intimate partner or another perpetrator, in the last 12 months were significantly associated with HIV/AIDS status. Again this is true both crudely and after the results were adjusted for variables (COR 2.4, 95% CI 1,4-4.4) and 1.4(1.0-2.1). This may be due to recent knowledge of HIV status (either by the woman or her partner has led to violence).

The more significant association between violence for the last 12 months when compared with life time partner violence could be attributed to different factors. One possible explanation may be all the last 12 months partner violence do occur with the existence of HIV epidemics, on the other hand the life time partner violence may occur during the epidemic of HIV and even before HIV epidemic. This can dilute the result and the association can be less than those partners violence within the last 12 months. The second possible explanation could be, the rapid spread of the epidemic in the rural community in the recent years, according to the study conducted by Ethiopian Public Health Association in 2005 [22]. The fact that the current study is focusing in the rural districts of South Wollo zone, the importance of last 12 month partner violence to associate more with HIV than the life time partner violence.

### Strengths and limitations of the study

#### Strength

The strength is related with representative site selection. The study area, South Wollo zone, has 21 districts. Of these, over 30% of the districts (seven), about 20% of VCT centers were included to get a representative sample for prevalence of sexual violence and HIV infection. The study has also included three remote districts, including Tenta, Legambo and Woreilu from the western part of South Wollo zone. The data collection was carried out by highly trained counselors who were working in the selected VCT centers.

### Limitations

The study lacks to feature adequate comparison, especially in associating violence and HIV, because of unavailability of similar studies. Similarly, there was a problem among respondents to recall some background and behavioral information about their respective partners. As a result, significant missing facts are observed in partner background and behavior. On top of these, there was under reporting of cases since both violence and HIV are sensitive issues.

## Conclusion

•Sexual violence is experienced by over one in three (36.3%) of the women involved in the study. Prevalence of HIV among women visiting health facilities is 21.5%. This study showed that there is a higher prevalence of sexual violence and HIV infection among illiterate women. Female education should, therefore, be promoted as a key strategy to tackle these gender related issues. In addition, the empowerment of women can be used as an important tool to prevent both sexual violence and HIV/AIDS. Since there is a significant association between sexual violence and HIV/AIDS, it is essential to provide counseling service on violence, for women who come seeking VCT. During VCT services, women who had experienced sexual violence should be referred to further counseling services.

•Based on the findings of the current study, attention should be given to young partners, especially to intimate and stranger male partners, to stop inflicting violent act on women in their respective communities. More importantly policy should be set to control mediating variables (women with multiple partners, women who chew chat, women with STI) that interact with violence to aggravate the transmission of HIV/AIDS in the community. Though the scope of the current study is limited to sexual violence, it is recommend that other types of violence that affect the health and empowerment of women should also get attention. Finally, according to the finding in tis study, the odds ratio between intimate partner violence for the last 12 month was found to be significant. In line with this, the attributable risk is found to be 18.5, this clearly demonstrates that violence on women needs an appropriate intervention policy.

## Methods

### Study area and period

This study was conducted in South Wollo zone, in Amhara Regional State of Ethiopia. South Wollo is one of the 10 zones in the Region. It is bordered in the South by North Showa zone, of Oromiya Region, in the Northwest by South Gondar, in the West by West Gojjam zone, of Amhara Region, in the North by North Wollo, in the Northeast by Afar Regional State, and in the East by Oromiya zone and Argoba special district.

Based on the 2007 census conducted by the Central Statistical Agency of Ethiopia (CSA), the Zone has a total population of 2,518,862, of whom 1,248,698 are men and 1,270,164 women. Even though there is no previous study conducted on HIV/AIDS and sexual violence, the situation of HIV/AIDS in the Amhara region is one of the worst in the country with persistently high prevalence particularly of the urban estimates. According to Ethiopian Demographic Health Survey (EDHS) 2011, The prevalence of HIV/AIDs in Amahara Region is 1.6%.

The study was conducted from February to April 2009 at 10 VCT centers of South Wollo zone.

### Study design

Facility based cross-sectional study was conducted at seven districts *(Legambo*, *Woreilu*, *Tewledere*, *Kutaber*, *Dessie*, *Worehimeno*, *Kalu***)** in South Wollo zone. Quantitative data was collected from 10 VCT centers, (health centers and hospitals), from the selected districts. The subjects were women of reproductive age, who were visiting health institutions seeking regular HIV counseling and testing, including antenatal care services. Four selecting criteria were used: a) all women who were examined for VCT b) able to communicate using the local Amharic language c) being permanent resident (living for a year or more or having an intention to live within the Zone) and d) willing to participate in the study.

### Sample size determination

To determine minimum sample size, the study used the figure 59% prevalence, of sexual violence, obtained from a previous study conducted in Meskan and Mareko district in central Ethiopia [13]. This study achieved 95% certainty, with maximum discrepancy of ± 4% between the sample and the total population. An additional 15% was added as a contingency to compensate for possible non- response. It was determined that a total of 667 women who use VCT in South Wollo zone were needed [24]. All eligible women who visited the selected VCT center of the Zone during the study period were included.

### Data collection procedures

The data were collected by 12 trained counselors from the respective selected VCT centers. The counselors were trained for two days on the questionnaire, eligibility criteria, confidentiality and other ethical issues. The data were collected using the standardized questionnaire from WHO-multi-country study on women’s health. The questionnaire was adapted to answer the main study questions of the research. It was translated into Amharic language and then back to English by the principal investigator and other personnel fluent in both languages, to prevent possible ambiguity. The questionnaire contained mainly closed questions, with a smaller proportion of open ended ones. It was pre-tested and necessary adjustments made on it.

Data were collected during the pre-test counseling before the HIV status of the respondents was known; the HIV status of the respondents was recorded after counseling. A total of 667 women were tested for HIV. Whole blood was taken from each study participant to detect the presence of HIV antibodies associated with HIV infection. The test was performed by using HIV rapid testing method, based on the National test algorithm. The National rapid test algorithm should use three rapid HIV test kits using the serial method. The algorithm uses three types of tests: the screening test, the confirmatory test, and a tiebreaker. The screening test is the first test in the sequence. The confirmatory test is used to confirm a positive result if the first test is positive. A tiebreaker is the final test which is done when there is a difference between the screening and confirmatory test results. In Ethiopia, we use KHB as a screening test, STAT-PAK as a confirmatory test, and Uni-gold as a tiebreaker.

If a test is non-reactive when using the KHB test kit, no other test was made and only reported as negative i.e. the individual tested was HIV negative. If the test is reactive with KHB, a second test was made using STAT-PAK to confirm the result. If the test was also reactive with STAT-PAK, it was reported as positive i.e. the individual tested was HIV positive. If the test is reactive with KHB, but non-reactive with STAT-PAK, then a tiebreaker test (Uni-gold) was carried out. If the Uni-gold is non-reactive, then the result was reported negative. However, if the Uni-gold is reactive, the result was reported positive [25].

The data collection process continued until the required amount of data, were collected. Two supervisors and the principal investigator monitored the data collection and checked responses to the questionnaire for consistency and completeness. (See the Additional file 1).

To assess the relationship between sexual violence and the risk of HIV infection, different variables were measured; these included socio-economic status, sexual violence history, and HIV status.

### Data quality

The quality of the data was assured by using a standardized, comprehensive questionnaire. The questionnaire was pre-tested before starting the actual data collection. Training was given for the counselors who completed the questionnaire. In order to facilitate open and honest communication with participants the majority of the counselors were females.

### Data analysis

Quantitative data was entered, cleaned and processed by SPSS (Statistical Package for Social Science). Cross tabulation for selected variables was conducted with the outcome variables (HIV status). Logistic regression was performed using SPSS (Statistical Package for Social Science version 10).

### Study variables

The dependent variable of the study is HIV status. The independent variable includes sexual violence, educational background, socio-economic status, demographic (age, marital status, parity and gravidity) characteristics, and their marital relationship with their spouses. For women who experienced sexual violence and who asked for counseling services the data were collected by the counselor in charge.

### Ethical clearance

Ethical clearance was obtained from, the research board of Faculty of Medicine, Addis Ababa University, before the start of the fieldwork. An official letter of co-operation was written to South Wollo zone administration. Informed consent was obtained from each client for participation in the study. HIV status of women was obtained anonymously, by VCT service providers. High degree confidentiality was maintained during data collection and, no name and other identifier were written on the questionnaire. Agreement was reached between the investigators and VCT centers in the area to provide the necessary counseling, care and support for HIV positive study subjects, including victims of violence.

## Appendix 1

### Sample size formula

Sample size formula: N= (Zα/_2_)^2^ p (1-p) /d^2^

N = Sample size

p= 0.59 (59%)

Zα/2 = 1.96

d=0.04 (4%)

Adding 15% for non response rate

(1.96)2 *.59(.41)/.(0.04) 2 = 580 + 15% none response

N= 667

## Abbreviations

AIDS: Acquired Immune Deficiency Syndrome; AOR: Adjusted odds ratio; ANC: Antenatal care; COR: Crude odds ratio; CI: Confidence interval; CSW: Commercial sex workers; DHS: Demographic Health Survey; EPHA: Ethiopian Public Health Association; EDHS: Ethiopia Demographic Health Survey; HIV: Human Immune Deficiency Virus; OR: Odds ratio; SPSS: Statistical Package for Social Science; USA: United State of America; VCT: Voluntary Testing and Counseling; WHO: World Health Organization.

## Competing interests

There is no competing interests between authors.

## Authors’ contributions

FH designed the data collection, analysis and prepared the draft thesis. Dr ND supervised the analysis and write up. Both authors read and approved the final manuscript.

## Supplementary Material

Additional file 1 Survey Questionnaire/Data collection instrument. Click here for file
