# Supplementary material for: The relationship between sexual violence and human immunodeficiency virus (HIV) infection among women using voluntary counseling and testing services in South Wollo Zone, Ethiopia
Source: BMC Res Notes. 2013 Jul 15;6:271. doi: 10.1186/1756-0500-6-271 (PMC3751471; doi:10.1186/1756-0500-6-271)
Supplement: Additional file 1 — Survey Questionnaire/Data collection instrument. [file 1756-0500-6-271-S1.doc]

Survey Questionnaire/Data collection instrument

Questionnaire, to assess sexual violence and the risk of HIVinfection in South Wollo Zone, Amhara National Regional State.

Questioner identification number __ Health institution Name ____

Checked by: Supervisor: Name Signature Date

Name of interviewer , Date of interview / /

Date Month Year

Part 1. Socio demographic data

| No | Questions | Coding categories |
| --- | --- | --- |
| 101 | How old are you? (in years) | _________ 1. Do not know ________2 |
| 102 | What is your current marital status? | (circle the response ) Single……..…………..…….. 1 Married…………………….....2 Widowed..................................3 Divorced……………..………4 Cohabiting ….…….…………5 |
| 103 | What is the highest educational level you completed? | Unable to read and write.….. 1 Able to read and write………..2 Completed grade ( ____ ) |
| 104 | What is your religion? | Orthodox ………….…………1 Muslim……………..…………2 Protestant ………….…………3 Others ( specify) ……….…….4 |
| 105 | What ethnic group do you Belongs to? | Amhara.....................................1 Oromo.......................................2 Others (specify)……………….3 |
| 106 | What is your current Occupation? | Government employee …………1 House wife ……………………..2 Others (specify) ………………..3 |
| 107 | Residence | Urban …………………………1 Rural…………………………….2 |
| 108 | Duration of living together with your current partner? | If it is less than one year write the month ( ) Number of completed years( ___ ) |
| 109 | Male partner's age in years | (…………) 1 Idont know…..2 |
| 110 | What is your partner’s educational status? | Unable to read and write ……1 Able to read and write----------2 Completed grade ( ____ ) |
| 111 | What is your partners current Occupation? | Government employee 1 Private employee ………………2 Daily laborer ………………….. 3 Driver …………………………..4 Merchant ……………………….5 Other (specifay)……………..….6 |
| 112 | Can you estimate your daily or monthly income in Birr | Daily ( ______ ) or Monthly (________ ) other …………. |
| 200 | Question on risk behavior for HIV | |
| 201 | Do you ever had sexual intercourse? | Yes………………………………1 No……………………………….2 I am not willing to answer…..…..3 |
| 202 | If your answer for question number 201 is yes what was your age when you first have sex? | (…………) …..1 Do not remember……..2 |
| 203 | If your answer for question number 201 is yes describe the situation of the sex. | I was willing to do it …………………1 I was unwilling to do it……………….2 I was forced to do it…………………..3 I do not remember…………………….8 Not willing answer……………………9 |
| 204 | If your answer for question number 201 is yes with how many people do you perform sex in your life time? | 1. One 2. Two 3. Three 4. Four 5. Five and above |
| 205 | In the past 12 months do you perform sex with your partner? | Yes…………1 No…………....2 Not willing to give answer….…9 |
| 206 | If your answer for 205 is yes do you use condom frequently? | Yes…………1 No…………....2 Not willing to give answer….…9 |
| 207 | If your answer for question number 206 is no why? | 1. Cannot get condom at the time 2. Lack of awareness 3. Because my partner do not want to use 4. Others __________________________ |
| 208 | In the past 12 months was there a fluid that comes out of your genital organ or do you have STD? | Yes…………1 No…………..2 Do not remember…………….8 Not willing to answer…..9 |
| 209 | If your answer for question number 208 is yes did you go to any health center? | Yes…………1 No…………....2 Not willing to give answer….…9 |
| 210 | If your answer for question number 208 is yes did you tell your partner to use condom? | Yes---------------1 no-------------2 Not willing to answer------9 |
| 211 | Age at first marriage (in years) (only for married girls) | ( )-----------1 do not know------2 |
| 212 | Do you chew chat? | Yes …………………………………1 No…………………………………..2 Not willing to answer………………9 |
| 213 | If your answer for 212 is yes how frequently? | Daily ……………….…………….1 1-3 days in a week…………………2 Sometimes………………………….3 Not willing to give answer…………..4 |
| 214 | Does your partner chew chat? | Yes …………………………………1 No…………………………………..2 Does not concern …………………3 Do not know………………………9 |
| 215 | If your answer is yes how frequently | Daily ……………….……………..1 1-3 days in a week…………..……2 Sometimes…………………..…….3 Not willing to give answer….……..9 |
| 216 | Do you drink alcoholic? | Yes …………………………..……1 No………………………..………..2 Not willing to answer…..…………9 |
| 217 | If your answer for 216 is yes how frequently? | Daily ……………….….………….1 1-3 days in a week…..……………2 Sometimes…………..…………….3 Not willing to give answer.………..9 |
| 218 | Does your partner drink alcoholi? | Yes ………………………..………1 No………………………..………..2 Does not concern …………………3 Do not know------………………….8 |
| 219 | If your answer for 218 is yes how frequently? | Daily ……………….…………….1 1-3 days in a week…………………2 Sometimes………………………….3 Do not know…………..8 |
| 220 | Do you discuss with your husband on matters related to | Financial Yes …..1 .No ……..2 .Family planning Yes …..1 .No ………..2 Sexuality Yes …..1 .No ………..2 HIV/AIDS Yes …..1 .No ..…..2 |
| 221 | Who is responsible in taking your children to hospital when they are sick? | Me………………………….………1 My husband…………………..…….2 Me and my husband……….……….3 Refused to answer…………….……9 |
| 222 | Who is responsible for buying smaller items in your house? | Me…………………….……………1 My husband…………….………….2 Me and my husband……………….3 Refused to answer…………………9 |
| 223 | Who is responsible for buying bigger items in your house like TV, ox? | Me…………………………………1 My husband……………………….2 Me and my husband……………….3 Refused to answer…………………9 |
| 224 | Did your husband ever hinder you from visiting your family or relatives? | Yes …………………………..1 No ……………………………2 Do not know…………………8 Refused to answer……………9 |
| 225 | Do you think your partner have relation with other girls before starting relation with you |  |
| 226 | If your answer is yes with how many girls? | 1 One 2, two 3. Three 4. Four 5. Five and above |
|  |  |  |
| 227 | Do you think your partner have relation with other girls after starting relation with you | Yes(with CSW)..........................1 Yes(Not with CSW)...................2 No ..............................................3 May be.......................................4 Do not know...............................8 Refused to answer……….........9 |
| 228 | If your answer is yes with how many girls? | 1. One 2, two 3. Three 4. Four 5. Five and above |
| 229 | If your answer for 227 is yes do you think he uses condom? | Yes ...............1 No....................2 . Do not know.........................3 |
| 230 | Reason for seeking HIV testing | Self initiated to know status…………..1 Was compulsory requirement…………2 Pre-marital………..………………….3 Required by physician ………………4 Other (Specify)……………………..5 |

Part 3 Questions related to gender

| 300 | Questions for sexual violence | | |
| --- | --- | --- | --- |
|  |  | **A. . Sexual abuse, life time** | **B .Sexual abuse, past 12 month** |
| 301 | Were you physically forced to have sex when you did not want to by your partner? | Yes …………………………..1 No ……………………………2 Never had sex…………………8 Refused to answer………….…9 | Yes ……………..…..1 No ………………..…2 Never had sex….…….8 Refused to answer……9 |
| 302 | If your answer is yes how many times? | One time…………………….1 Few times……………………2 Many times…………………..3 | One time…………….1 Few times……………2 Many times…………..3 |
| 303 | Has anyone else outside your partner forced you to do something sexual that you found degrading or humiliating? | Yes …………………………..1 No ……………………………2 Never had sex…………………8 Refused to answer……………9 | Yes ……….………1 No ………….……..2 Never had sex………8 Refused to answer…9 |
| 304 | If your answer is yes how many times? | One time…………………….1 Few times……………………2 Many times…………………..3 | One time…….……….1 Few times……………2 Many times…………..3 |
| 305 | Were you forced to have sex when you did not want because you were afraid of what your partner might do to you? | Yes …………………………..1 No ……………………………2 Never had sex…………………8 Refused to answer……………9 | Yes ………………..1 No…………….……2 Never had sex……….8 Refused to answer….9 |
| 306 | If your answer is yes how many times? | One time…………………….1 Few times……………………2 Many times…………………..3 | One time……………….1 Few times………………2 Many times…..………..3 |
| 307 | Has your partner forced you to do something sexual that you found degrading or humiliating? | Yes …………………………..1 No ……………………………2 Never had sex…………………8 Refused to answer……………9 | Yes ……….………1 No ………….……..2 Never had sex………8 Refused to answer…9 |
| 308 | If your answer is yes how many times? | One time…………………….1 Few times……………………2 Many times…………………..3 | One time……………….1 Few times………………2 Many times……………..3 |
| 309 | Have you faced rape in your life time? | Yes …………………………..1 No ……………………………2 Do not know………………….8 Refused to answer……………9 | Yes ………………..1 No …………….……2 Do not know.………8 Refused to answer.…9 |
| 310 | How was your marriage arrangement? | By family ……………………..1 By agreement…………………2 By abduction…………………3 Doesn’t concern me…………..4 Do not know…………………8 Refused to answer……………9 |  |
| 311 | Before the age of 15, do you remember if any one in your family ever touched you sexually, or made you do something sexual that you didn’t want to? 1 Yes 2. No IF YES: Who did this to you? | Father…………………………1 Step father……………..……2 Family member………….….3 Teacher…………………….…4 Police/solider ………………. 5 Boy friend……………………6 Stranger………………………7 Other ………………………..8 |  |
| 312 | Is it a wife’s obligation to have sex with her husband even if she does not feel like it? | Agree. ...............................1 Disagree........................... 2 Do not know.....................8 Refused no answer...........9 |  |
| 313 | In your opinion, can a married women refuse to have sex with her husband if: | Yes.......................1 No ........................2 Do not know ........9 |  |
| 314 | If your answer is yes in what situation can she refuse? | A. she does not want B. he is drunk C. she is sick D. he mistreat her | Yes No DK 1 2 8 1 2 8 1 2 8 1 2 8 |
| 400 | **KAP on modes of Transmission and Prevention** | | |
| 401 | If yes, what are the modes of transmission of HIV? [Record all possible answers] | Unprotected sex …………………1 From mother to her baby………..2 Through blood transfusion………3 Contaminated instruments………..4 Eating together with PLH………..5 Through mosquito bit………………6 Do not know……………………….8 | |
| 402 | what are the modes of prevention of HIV? [Record all possible answers] | 1 Abstain form sex 2 Be faithful to only one partner 3 Use condom 4. Avoid unsafe blood transfusion 5. Not share sharp things 6) Avoid mosquito bites 7 Avoid eating with PLH 8. do not know | |
| 403 | HIV status | Positive ………….1 Negative…………….2 | |

Thank you for your time.
